# Supplementary material for: Seminoma and Embryonal Carcinoma Footprints Identified by Analysis of Integrated Genome-Wide Epigenetic and Expression Profiles of Germ Cell Cancer Cell Lines
Source: PLoS One. 2014 Jun 2;9(6):e98330. doi: 10.1371/journal.pone.0098330 (PMC4041891; doi:10.1371/journal.pone.0098330)
Supplement: File S1 — ZIP file containing DMRforPairs output for significant regions. Please start from the html files. (ZIP) [file pone.0098330.s008.zip › figures/3921.pdf]

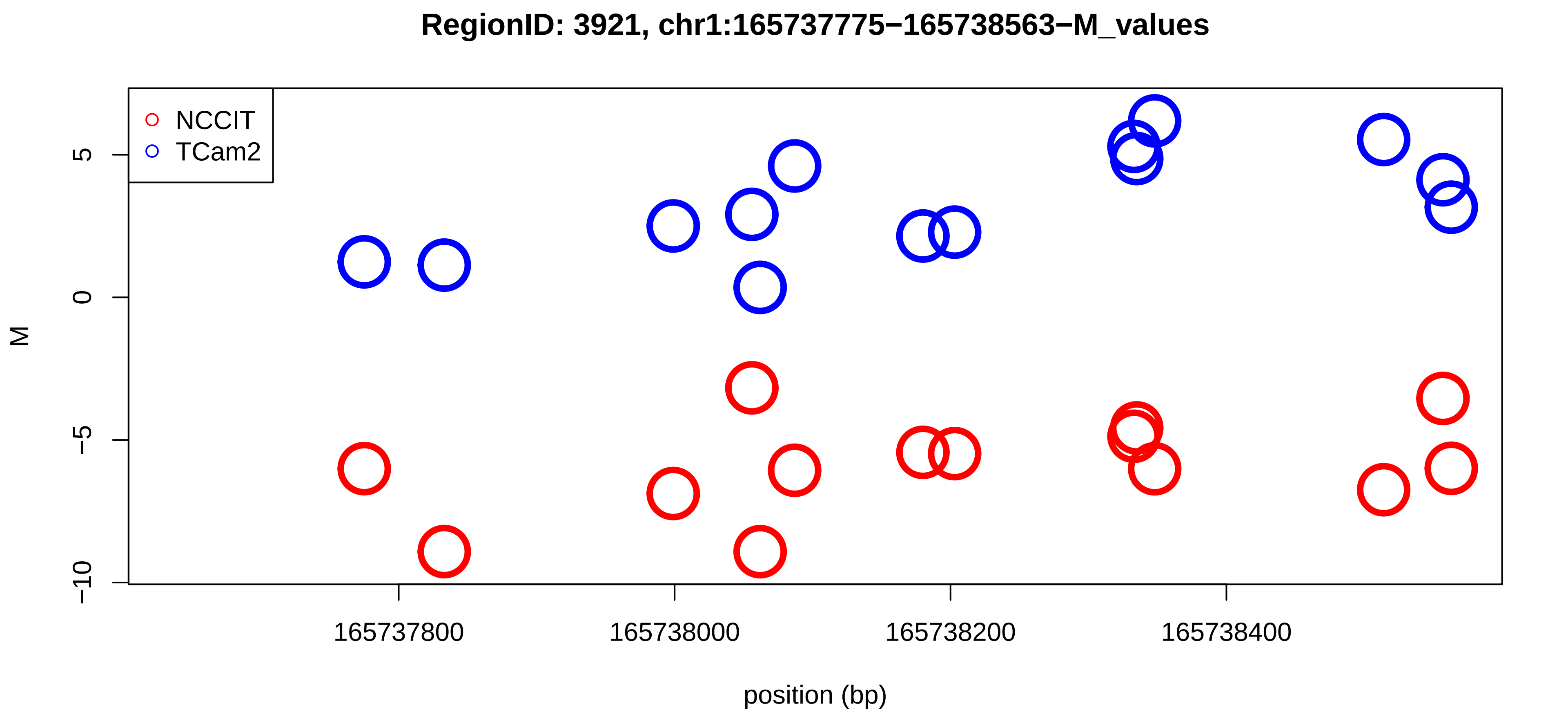

RegionID: 3921, chr1:165737775−165738563−M_values
NCCIT
TCam2
M
5
0
−5
−10
165737800
165738000
165738200
165738400
position (bp)

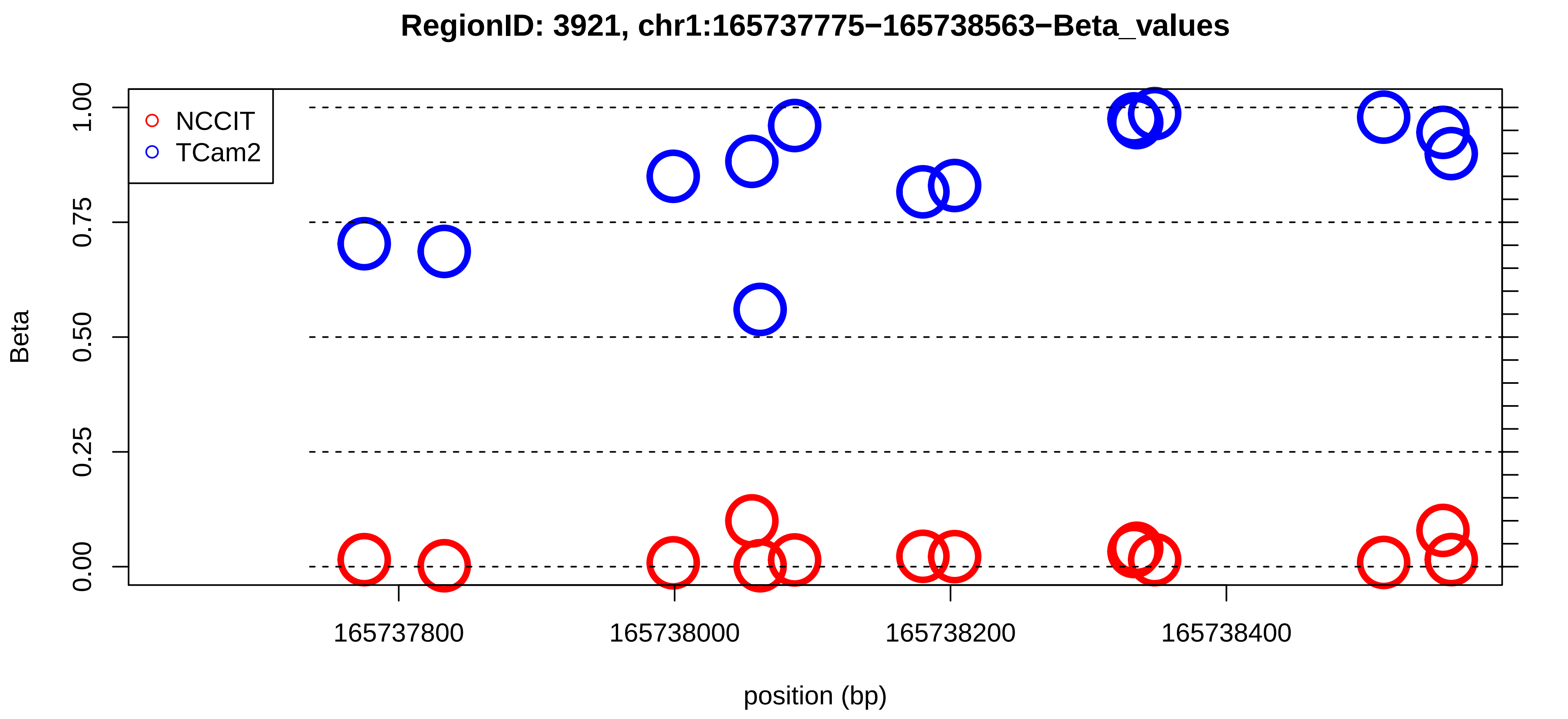

RegionID: 3921, chr1:165737775−165738563−Beta_values
NCCIT
TCam2
Beta
1.00
0.75
0.50
0.25
0.00
165737800
165738000
165738200
165738400
position (bp)

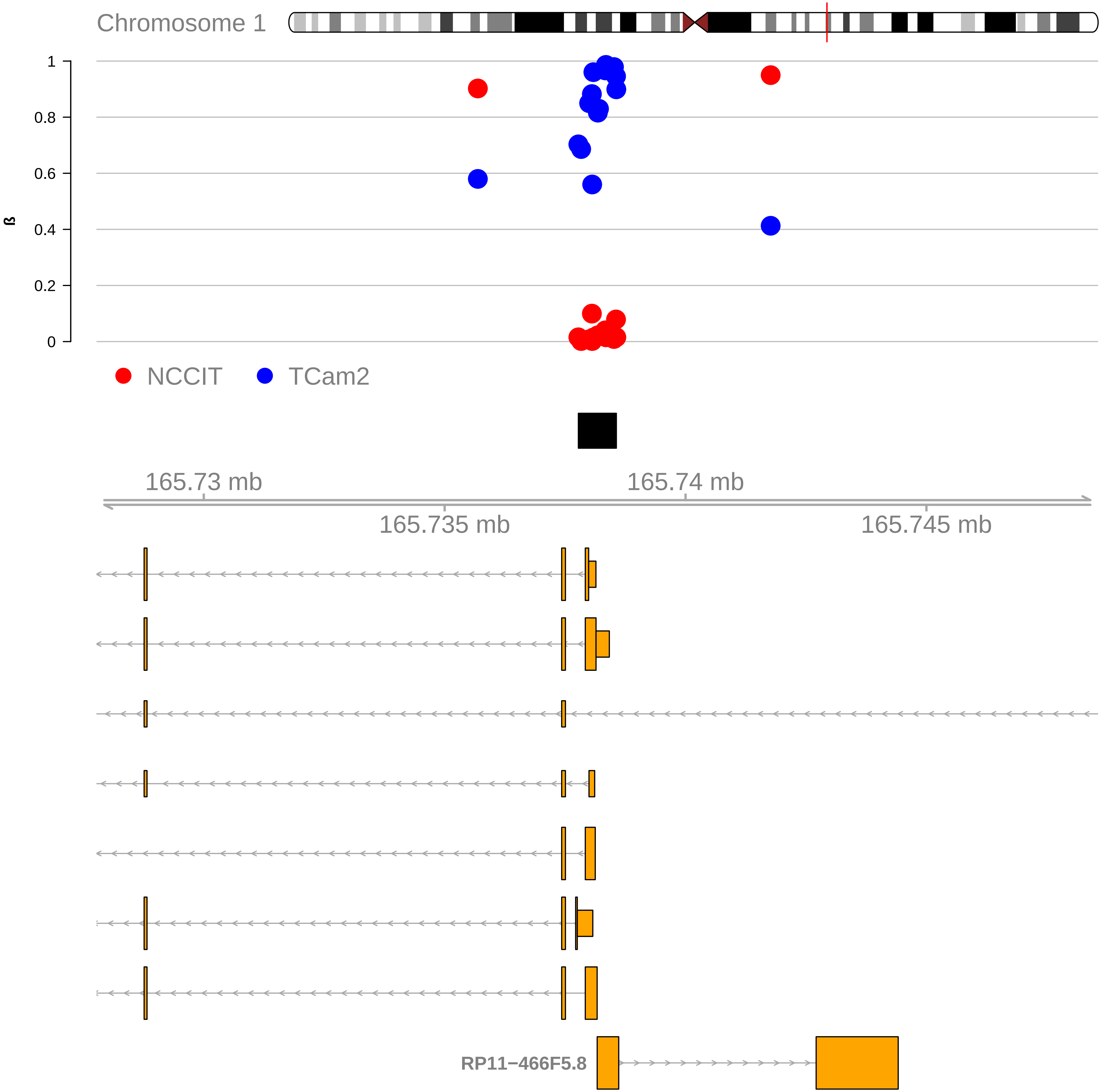

Chromosome 1
1
0.8
0.6
0.4
0.2
0
ß
NCCIT
TCam2
165.73 mb
165.74 mb
165.735 mb
165.745 mb
RP11−466F5.8
